# DeepCMS: A Feature Selection-Driven Model for Cancer Molecular Subtyping with a Case Study on Testicular Germ Cell Tumors

**DOI:** 10.3390/diagnostics15212730

**Published:** 2025-10-28

**Authors:** Mehwish Wahid Khan, Ghufran Ahmed, Muhammad Shahzad, Abdallah Namoun, Shahid Hussain, Meshari Huwaytim Alanazi

**Affiliations:** 1 Department of Computer Science, National University of Computer and Emerging Sciences, Karachi 75030, Pakistan; mehwish.wahid@nu.edu.pk (M.W.K.); ghufran.ahmed@nu.edu.pk (G.A.); 2 Department of Computer Science, GU Tech, Al Ghazali University, Karachi 75340, Pakistan; muhammad.shahzad@agu.edu.pk; 3 AI Center, Faculty of Computer and Information Systems, Islamic University of Madinah, Madinah 42351, Saudi Arabia; 4 Department of Computer Science and Software Engineering, Penn State University, Behrend, Erie, PA 16563, USA; shussain@psu.edu; 5 Computer Science Department, College of Sciences, Northern Border University, Arar 73213, Saudi Arabia

**Keywords:** deep learning, feature selection, cancer molecular subtyping, data scarcity, multi-omics potential

## Abstract

**Background/Objectives:** Cancer is a chronic and heterogeneous disease, possessing molecular variation within a single type, resulting in its molecular subtypes. Cancer molecular subtyping offers biological insights into cancer variability, facilitating the development of personalized medicines. Various models have been proposed for cancer molecular subtyping, utilizing the high-dimensional transcriptomic, genomic, or proteomic data. The issue of data scarcity, characterized by high feature dimensionality and a limited sample size, remains a persistent problem.The objective of this research is to propose a deep learning framework, DeepCMS, that leverages the capabilities of feed-forward neural networks, gene set enrichment analysis, and feature selection to construct a well-representative subset of the feature space, thereby producing promising results. **Methods:** The gene expression data were transformed into enrichment scores, resulting in over 22,000 features. From those, the top 2000 features were selected, and deep learning was applied to these features. The encouraging outcomes indicate the efficacy of the proposed framework in terms of defining a well-representative feature space and accurately classifying cancer molecular subtypes. **Results:** DeepCMS consistently outperformed state-of-the-art models in aggregated accuracy, sensitivity, specificity, and balanced accuracy. The aggregated metrics surpassed 0.90 for all efficiency measures on independent test datasets, showing the generalizability and robustness of our framework. Although developed using colon cancer’s gene expression data, this approach may be applied to any gene expression data; a case study is also devised for illustration. **Conclusions:** Overall, the proposed DeepCMS framework enables the accurate and robust classification of cancer molecular subtypes using a compact and informative feature set, facilitating improved precision in oncology applications.

## 1. Introduction

The application of artificial intelligence in biomedical big data has gained considerable prominence in recent years. Several studies have utilized biomedical data to explore various aspects, including disease classification [1], prediction models based on sensor data [2], predicting drug target interaction [3,4], and disease prognosis [5], etc. Specifically, deep learning (DL) and machine learning (ML) have demonstrated outstanding results in various healthcare domains. Healthcare encompasses a wide range of domains, including medical IoT [6], electronic health record (EHR) data [7], and OMICs data-driven disease diagnosis, prognosis, management, patient outcomes, disease types, and subtype classification [8]. DL has become essential for drug discovery and personalized medicine, as it sifts through vast cell lines and drugs. DL has been extensively applied to cancer research, with ongoing efforts to examine the disease from various aspects. Cancer is a complex and deadly disease that often becomes resistant to medication [9]. Multiple genetic factors contribute to its development and progression. Cancers are categorized based on the organ where they originated, like breast cancer, lung cancer, or skin cancer [10]; however, heterogeneity exists even within the same cancer type. The distinct molecular and clinical behaviors observed within the same cancer type are categorized as molecular subtypes [11]. Different molecular subtypes lead to variable patient responses; knowing the subtype can help select personalized therapies for patients and develop new targeted drugs. Advancements in the field of next-generation sequencing have substantially accelerated the growth of research in various diagnostic aspects, including cancer molecular subtyping. The enhanced accessibility of genome-wide multi-omics data has created an unprecedented prospect for exploring cancer molecular subtypes. The multi-OMIC data provide insight into the molecular variation, which may be highly valuable in precision medicine [12]. Various data types, including histopathological images, transcriptomic data, and genomic data, are utilized to predict cancer subtype.

Transcriptomic data provide valuable molecular insights, though its usefulness is often hindered by the small sample size as compared to its high dimensionality. This research aims to achieve high accuracy while utilizing fewer dimensions. In this work, the expression data are transformed into enrichment scores for each sample and gene set pair. Following that, the top 2000 features (the gene sets) are selected. After solving the class imbalance issue, the model is trained using the best-selected features. The model is then tested on four validation datasets, which were not part of the training cohort. The proposed approach has shown better aggregated results on multiple datasets. To validate the robustness of the proposed framework, different efficiency measures like sensitivity, specificity, balanced accuracy, receiver operating characteristic (ROC) curve, and area under the curve (AUC) were evaluated. Model performance was evaluated using classification outcomes—true positives *(TP),* true negatives *(TN)*, false positives *(FP),* and false negatives *(FN)*—and the derived measures true positive rate (TPR) and false positive rate (FPR). The following are the mathematical expressions for all these metrics.

(1) $$\mathrm{Accuracy}=\frac{TP+TN}{TP+TN+FP+FN}$$

(2) $$\mathrm{Sensitivity}=\frac{TP}{TP+FN}\times 100$$

(3) $$\mathrm{Specificity}=\frac{TN}{TN+FP}\times 100$$

(4) $$\mathrm{Balanced}\;\mathrm{Accuracy}=\frac{1}{2}\left(\frac{TP}{TP+FN}+\frac{TN}{TN+FP}\right)$$

(5) $$\mathrm{AUC}=\int_{0}^{1}\mathrm{TPR}\left(\mathrm{FPR}\right)\;d\left(\mathrm{FPR}\right)$$

The novelty of our work and the contribution in this paper can be summarized as follows.

1. The proposed methodology addresses one of the major issues faced by the research community working with genomic or transcriptomic data: data scarcity, i.e., the significant disparity between the number of samples and the number of features.
2. The robustness of the proposed approach was assessed by comparing it with the state-of-the-art models. Its superiority over other state-of-the-art models, including Random Forest, SVM, and DeepCC, in terms of aggregated accuracy, aggregated sensitivity, aggregated specificity, and aggregated balanced accuracy is clearly demonstrated.
3. To evaluate the generalizability of the framework, a case study was designed using another cancer type: testicular germ cell tumor (TGCT). The same pipeline was followed on the TGCT dataset. Comparative results were also obtained on this dataset, achieving an accuracy of 0.97.

### Related Work

Cancer is a disease caused by a genetic disorder, having phenotypic and genetic variability among its different types and even within the same cancer type. The molecular subtype of cancer refers to a cohort that possesses similar molecular and clinical characteristics. A variety of molecular data, including gene expression profiling, mutation profiles [13], copy number variation (CNV), miRNA expression [14], and DNA methylation [15], have been employed for this purpose. Table 1 summarizes the major contributions in this field.

Classifying the molecular subtypes of breast cancer, a model combining the gene expression data with copy number variation (CNV) and histopathological images was designed. They used a convolutional neural network for the image dataset and a deep neural network for CNV and gene expression data. They combined the outputs from both at the end using weighted linear aggregation  [16]. Utilising the H and E slides  [17], tiles of different resolutions were generated, 2.5×, 5×, and 10×, and  an Inception Resnet-based CNN architecture for the prediction of histological and molecular subtypes of endometrial cancer. Each resolution tile was processed in a separate Inception-ResNet-based branch until the third-last layer, where all three were concatenated to produce the final prediction. It produced good results, although multi-resolution models could be computationally extensive. Using the ultrasound images, a ResNet50 model was trained as the base architecture to predict the breast cancer molecular subtypes  [18]. Stochastic Gradient Descent (SGD) was used as the optimizer, and data augmentation was also applied to each image to prevent overfitting. Their accuracy ranged from 80 percent to 97 percent for the first test dataset and 87 percent to 98 percent for the second test cohort.

In their work [19], they employed a deep learning model to perform cancer molecular subtyping utilizing gene coexpression networks to identify network features for each sample. The gene expression data were transformed into gene coexpression networks specific to each subtype, from which the particular modules were identified. Then the network features were identified, which were fed into the DNN for the classification of subtype. To reduce the large feature space of the multi-OMICS data, various approaches have been designed. Denoising autoencoders are used to generate combined and low-dimensional features from multi-OMICs data  [20]. The transformed low-dimensional data were then clustered into subtypes. The clustered subtype data were used to build a lightweight logistic regression classification model using the mRNA data to predict the subtype of ovarian cancer. RNAseq data from lung adenocarcinoma, along with the clinical information, were utilized for the classification of molecular subtypes of pyroptosis. Consensus clustering was employed to identify the pyroptosis-related molecular subtypes, which were subsequently used for the classification. The classification model was designed using the CatBoost [21].

Utilising multi-omics data, an adversarial learning framework was designed by employing a multiple-input multiple-output neural network architecture  [22]. Initially, features were extracted from each of the omics data. A subtype GAN model was then used to learn the differences and similarities among various omics data. Consensus clustering in conjunction with a Gaussian mixture model, was employed to identify the molecular subtypes of the tumor samples.

In some studies, including [23], authors integrate copy number variation (CNV) and gene expression profiles in the context of cancer molecular subtyping. Such integration does provide a comprehensive insight into the molecular characterisation, though it presents specific challenges, including computational complexity, data heterogeneity, and normalisation issues. The classification of breast cancer molecular subtypes was performed using whole slide image(WSI) tiles. Two different classifiers were designed, one for tumour and non-tumour classification and the other for molecular subtyping. The WSI was tiled into a 512 size and used for molecular subtype analysis. The tiles were classified as tumour or non-tumour; then, only the tumour tiles were used for molecular subtyping [24]. A combined approach was designed, utilizing a convolutional neural network in conjunction with a bidirectional gated recurrent unit (BiGRU). The BiGRU analyzes the deep features while retaining important information, and the CNN extracts the local features. Their model presents good accuracy, although it has only utilized gene expression data [25]. A supervised deep learning framework is designed for colorectal cancer subtyping. They considered the already known subtypes of CRC, CMS1-CMS4 [26]. Subtype-specific genes identified through fold change analysis were used as input to the deep learning model, enabling more accurate classification of cancer subtypes.

The researchers explore the multimodality nature of the data. Magnetic Resonance Imaging (MRI) images have also been utilized for the prediction of a 3-group classification of lower-grade glioma molecular subtypes. Using multimodality data [27], they included the numeric patient’s profile data, such as age, gender, tumor position, calcification in the CT, and the tumor to normal brain uptake (T/N) ratio, along with the MRI image data. They achieved a high training accuracy of 97.4, though on the testing dataset, the maximum they could reach was 68 percent. For the prediction of muscle-invasive bladder cancer molecular subtypes, the authors in [28] used the histomorphological slides and trained a residual neural network (ResNet) model. Their results, when compared with those of human pathologists, were found to be superior to those of human experts. Although molecular subtyping based on images has produced promising results, the inherent heterogeneity of cancer indicates that tumors with similar appearances may exhibit different clinical behaviors and variable drug responses [29].

## 2. Materials

### 2.1. Datasets

The TCGA gene expression dataset (*n* = 558) was used for model training. Samples without class labels (*n* = 108) were excluded, and only labeled data were retained for analysis. The resulting 450 samples were distributed across the four molecular subtypes as 68, 204, 63, and 115, respectively. The data reflected the class imbalance, which was resolved using the Synthetic Minority Oversampling Technique (SMOTE) [30], resulting in an equal distribution of 204 samples of each class. Oversampling was conducted using Python 3.9.7 in Jupyter Notebook 6.4.5, distributed through Anaconda 22.9.0 (Anaconda, Inc., Austin, TX, USA). Jupyter Notebook is an open-source environment developed by Project Jupyter (Berkeley, CA, USA). The Synthetic Minority Over-sampling Technique (SMOTE) (Tampa, Florida, United States) was implemented using the imbalanced-learn (imblearn) package, version 0.12.0. Oversampling was performed before the feature selection step, specifically on the training dataset. For model validation, the public datasets from the Colorectal Cancer Subtyping Consortium (CRCSC) [31], GSE39582, GSE13067, GSE37892, and GSE17536 were utilized. Together, four consensus molecular subtypes of colon cancer are employed [32], such as CMS1, CMS2, CMS3, and CMS4. The training and testing data are downloaded from the Synapse official website [33], (accessed on 26 March 2024). For the case study of testicular germ cell tumor (TGCT), the TCGA dataset is used, downloaded from the BioCportal website. The dataset originally contained 149 samples, of which 144 labeled data were used for training and validation. It is based on data generated by The Cancer Genome Atlas (TCGA), which is managed by the NCI and NHGRI. Information about TCGA can be found at [34].

### 2.2. Methods

**Figure 1:**
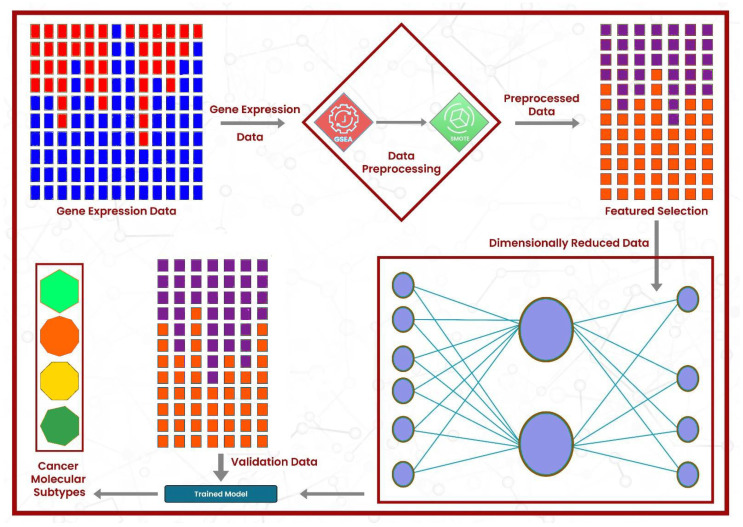
The architectural design of the proposed framework: the gene expression data first undergo enrichment analysis, followed by the resolution of class imbalance using SMOTE. Then, the best features are selected, and model training is performed.

Gene Set Enrichment Analysis (GSEA) [35] was applied to the gene expression data, leveraging the MSigDB v7 database via R, version 4.3.3 (R Foundation for Statistical Computing, Vienna, Austria) package DeepCC [36], version 0.1.1. The enrichment analysis resulted in 22,596 features, where each cell represents the enrichment score of the sample in a particular gene set. From these 22,596 features, the best 2000 features were selected using the Python scikit-learn library’s (version 1.5.0; Scikit-learn Developers, INRIA, Saclay, France) function ’SelectKBest()’, keeping f-regression as the scoring metric. To classify the cancer molecular subtype of a given sample, we utilized a fully connected feed-forward neural network model, DeepCC, due to its high accuracy. Its architecture consists of five dense layers, where the first layer has 1024 neurons, followed by 256, 64, 64, and 10 neurons. SELU was used as the activation function in all layers except the output layer, which utilized the Softmax function activation function for molecular subtype classification. The model was implemented in R using the Keras 2.15.0 and TensorFlow 2.16.0 packages (Google LLC, Mountain View, CA, USA) within RStudio/2024.04.1+748 (Posit Software, PBC, Boston, MA, USA). Each dense layer was followed by a batch normalization layer, followed by a Gaussian dropout (rate = 0.4), to minimize the overfitting. The model used the Adam optimizer, with a learning rate of 0.001 and a weight decay of 1 $\times \;10^{-6}$, and employed categorical cross-entropy loss. The model is trained using the top 2000 features of the TCGA training dataset and then validated using the separate CRCSC datasets. The validation sets were also reduced to the same two thousand features as the training cohort. Figure 1 graphically summarizes the methodology of this work. To evaluate the proposed model, it was compared with the state-of-the-art models, including DeepCC, Random Forest, and Support Vector Machine (SVM), using the default parameters in *R*. The entire pipeline was executed in *R*, except the oversampling and feature selection steps, which were executed in *Python*. As shown in Algorithm 1, the proposed DeepCMS framework integrates gene enrichment analysis, feature selection, and deep learning to effectively classify cancer molecular subtypes.
**Algorithm 1** DeepCMS**Input:** Gene expression data**Output:** Cancer molecular subtype prediction  Algorithm Steps:1:Perform the GSEA on the expression profile and calculate the enrichment score (ES) for each gene set in every sample.(6)$$\begin{array}{ccccccc} P_{\mathrm{hit}}\left(S,i\right),= & =\sum_{g_{j}\in S\;j\leq i}\frac{|r_{j}{|}^{p}}{N_{R}},= & \mathrm{where} & N_{R} & = & \sum_{g_{j}\in S} & {\left|r_{j}\right|}^{p} \end{array}$$
(7)$$\begin{array}{cc} P_{\mathrm{miss}}\left(S,i\right) & =\sum_{g_{j}\notin S\;j\leq i}\frac{1}{\left(N-N_{H}\right)}. \end{array}$$2:Select the 2000 best gene sets (features) from the 22,596 gene sets using the SelectKbest()3:Train the deep neural network model using those 2000 features.4:Validate the model using a different dataset.

## 3. Results

**Figure 2:**
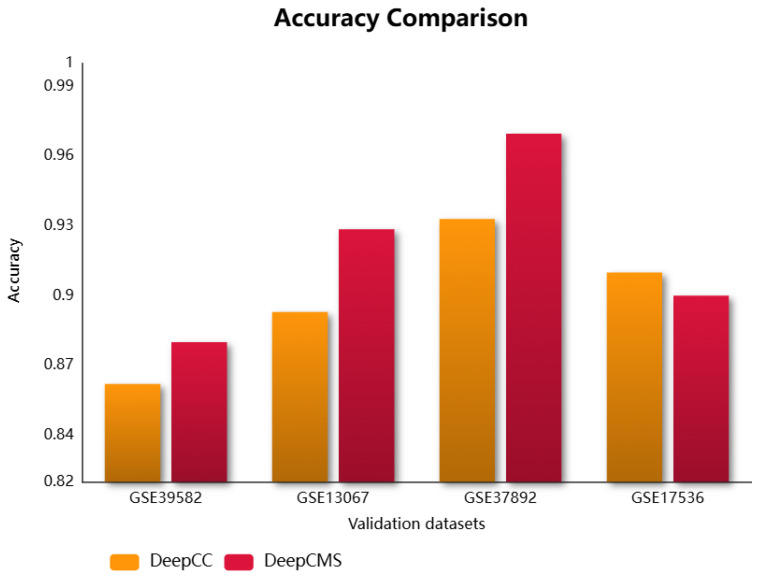
Comparison of the accuracy of DeepCMS and DeepCC across different validation datasets.

Molecular subtypes group patients with similar molecular and clinical behaviors, which assists in designing personalized treatments, drug targets, and response prediction. This research utilizes deep neural networks for predicting the molecular subtype of cancer. DeepCMS represents a significant advancement in producing better and more accurate results while using fewer features. Its classification aggregated accuracy compared with the state-of-the-art models, Random Forest, SVM, and DeepCC, was found to be superior. The trained model was validated using four microarray colon cancer datasets curated by CRCSC. The proposed framework outperformed the existing model, DeepCC, in three out of four datasets, with the fourth dataset also showing comparable performance. The comparison of different efficiency measures for DeepCMS and DeepCC is summarized in Table 2 and graphically represented in Figure 2. DeepCC is a deep learning-based model for cancer molecular subtype classification, demonstrating high performance. It utilizes the enrichment scores for model training, extracted from the gene expression data. In this study, we aim to achieve higher accuracy by leveraging its architecture with a reduced feature set. As demonstrated in our results, the proposed approach attains improved performance as compared to the original model.

**Figure 3:**
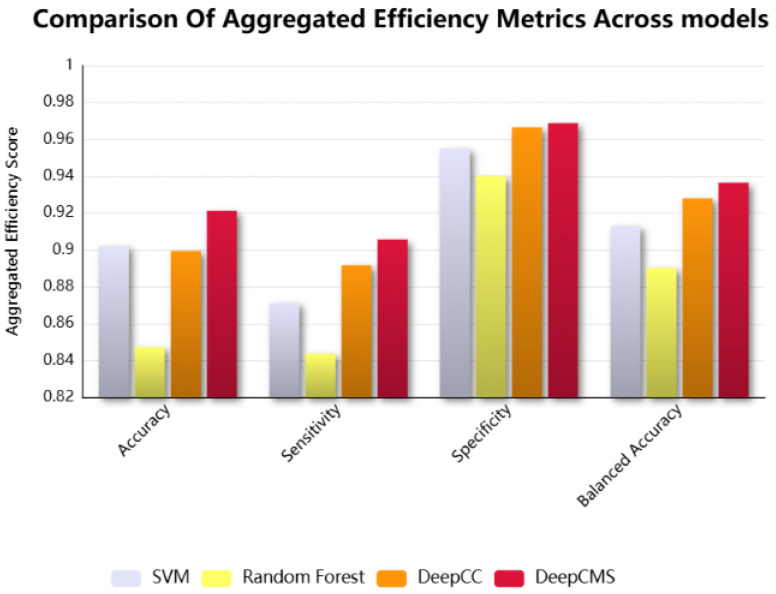
The comparison among the representative efficiency measures, including accuracy, sensitivity, specificity, and balanced accuracy of the proposed framework, DeepCMS, with the state-of-the-art models, SVM, Random Forest, and DeepCC.

DeepCMS shows superior aggregated results over multiple efficiency metrics. The comparison of different efficiency measures, such as sensitivity, specificity, and balanced accuracy, between the proposed model and the state-of-the-art models, Random Forest, SVM, and DeepCC, is graphically shown in Figure 3. Our model surpasses the existing models in key efficiency measures, including accuracy, sensitivity, specificity, and balanced accuracy.

**Figure 4:**
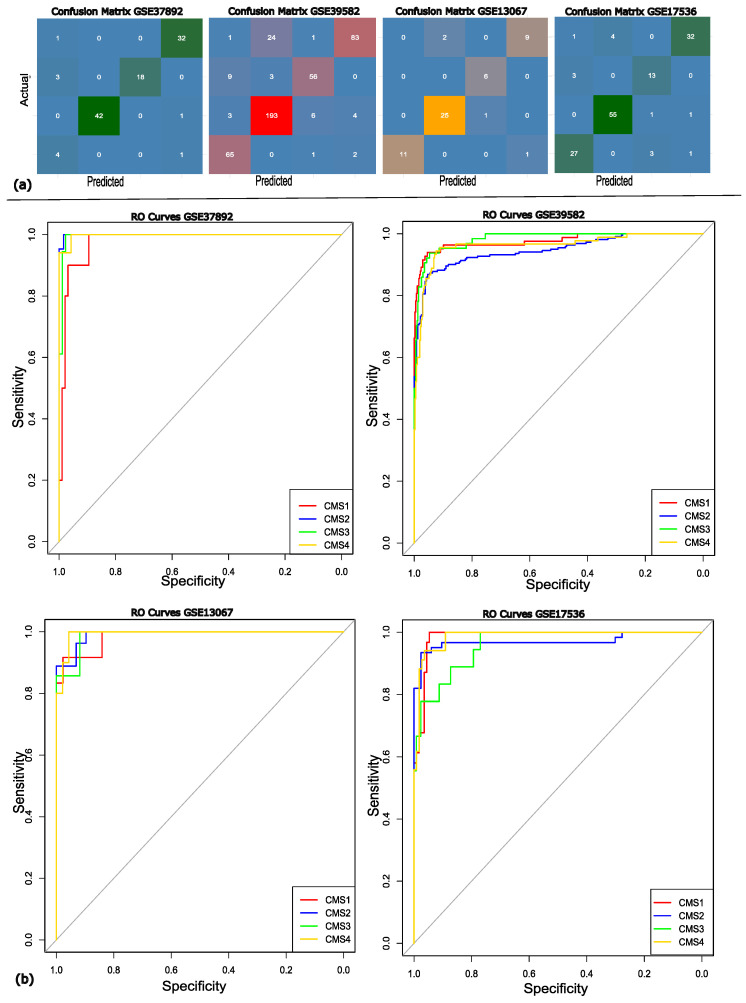
(**a**) The confusion matrix for each of the validation datasets. (**b**) The RO curves showing the efficiency of each of the datasets, where the red, blue, green, and yellow lines represent the different molecular subtypes of colon cancer; CMS1, CMS2, CMS3, and CMS4, respectively. The grey diagonal line represents the random classifier’s (AUC = 0.50) performance.

The performance of the classification model was further evaluated by calculating the area under the curve (AUC) for each validation set separately. The AUC for the validation dataset GSE37892 was 0.9975 with the confidence interval (CI) of 95%, the AUC for GSE13067 was 0.987 (95% CI), the AUC of GSE39582 was 0.9682 (95% CI), and GSE17536 got the AUC of 0.986 (95% CI). The above AUC values demonstrate that our model exhibits classification proficiency and is capable of distinguishing between the various molecular subtypes of the disease. The confusion matrix and ROC curves are illustrated in Figure 4.

## 4. Discussion

Cancer is a leading cause of mortality globally. Generally, one in every six deaths worldwide is attributable to cancer [37]. It is a complex disease driven by various genetic and phenotypic determinants that shape specific cancer types. Even within the same cancer type, multiple tumours are molecularly heterogeneous, resulting in variable drug response and clinical symptoms. The molecular subtype of cancer refers to a cohort possessing similar molecular and clinical behavior. A variety of molecular data, such as gene expression profiling, mutation profiles, copy number variation (CNV), miRNA expression, and DNA methylation, have been employed for this purpose. Cancer molecular subtyping is a critical step towards personalized medicine, identifying drug targets, and also predicting drug response. It provides insight into the molecular similarity within a subtype and the heterogeneity among the subtypes. High-throughput methods have facilitated more accurate molecular classification using OMICS data; specifically, RNA-seq-based cancer subtyping offers valuable insights by incorporating epigenetic and tumor microenvironmental factors, as well as intrinsic cellular properties, which underlie tumor heterogeneity [38]. Though proteomics and other multi-omics subtyping schemes also provide accurate cancer heterogeneity classification, they are more costly and resource-intensive and require specialized expertise as compared to RNAseq, specifically gene expression-based classification, which has emerged as a successful method [39].

Gene expression data refers to the biological information used to uncover significant patterns within gene datasets. This type of information is crucial for disease diagnosis, prognosis, and in evaluating the drug response. Expression data is high-dimensional, though it lacks sample size. To overcome the high dimensionality and low sample size problem, various approaches are employed, such as data augmentation, where the training sample size is increased or decreased using different techniques to minimize overfitting. To address the high dimensionality issue in the dataset, two distinct approaches are utilized: feature selection and feature extraction. The former selects the best features from the datasets by calculating the correlation of each feature with the target and then converting it to an F-score, and then selecting the features with a high F-score. In contrast, the latter extracts new features from the original dataset features. Both are preprocessing techniques used for dimensionality reduction. We wanted to retain the original features and employed the feature selection approach. Analyzing gene expression levels in isolation is often insufficient for a comprehensive understanding of complex diseases, such as cancer. To gain a more comprehensive perspective, advanced techniques have been developed that enable the simultaneous examination of multiple genes while integrating these data with other biological information. These integrative approaches facilitate a deeper exploration of the underlying molecular mechanisms and contribute to a deeper understanding of disease pathology. Gene expression profiles, transformed into pathway activities, have proven to be more informative and reliable for disease classification [40]. The gene expression data first undergo enrichment analysis (GSEA) [35], and the enrichment scores for each sample across all gene sets are utilized for training. Following this transformation, the data include 22,596 features with a few hundred samples. To minimize this overhead, the proposed framework first applies feature selection to select the best two thousand features, which are then used as the training cohort. When compared with the state-of-the-art models DeepCC, Random Forest, and SVM, it was found that the proposed approach showed better accuracy in the majority of datasets. Our approach yielded superior aggregated results in terms of accuracy, sensitivity, balanced accuracy, and specificity when compared to DeepCC, Random Forest, and SVM. This highlights the robustness and the generalizability of the proposed approach despite dataset-specific variations. DeepCC is a state-of-the-art model that accurately classifies the cancer molecular subtypes. For this, it utilizes more than 22,500 features; using such a high-dimensional feature space demands high computational complexity. The focus of this study was to produce better results while reducing the feature space. A comparison between the proposed approach and the state-of-the-art DeepCC model across four primary efficiency measures–accuracy, sensitivity, specificity, and balanced accuracy, is summarized in Table 2. The findings demonstrate that the proposed approach surpasses DeepCC across three validation datasets and achieves a comparable level of accuracy in the fourth. Accuracy represents the overall percentage of the correctly classified samples; sensitivity and specificity, respectively, reflect the rates of correctly identifying positive and negative cases. It is evident in the results presented in Table 2 that our approach attains superior accuracy, indicating the classification strength of the proposed model. Although performance on one dataset was slightly lower than that of DeepCC, the aggregated results across datasets highlight that our model consistently outperforms existing methods when evaluated across multiple efficiency criteria. The enhanced aggregated sensitivity and specificity of the proposed model highlight its strength in discriminating between subtypes, enabling more accurate identification of true positives and true negatives. The aggregated results are summarized in Table 3 and graphically represented in Figure 3. These findings underscore both the effectiveness and generalizability of the proposed approach.

It can be observed in the confusion matrix heatmaps that the model shows high positive ratings across all subtypes. However, more frequent subtype CMS3 misclassifications were predicted as CMS1, which may be attributed to their overlapping molecular signatures. The robustness of the model was further supported by the ROC curve analysis, with AUC values exceeding 95. The ROC analysis and confusion matrix both reflect that the proposed approach achieves strong overall discriminative power. To conclude the discussion, the proposed approach represents superior performance compared to existing models across multiple datasets, highlighting its robustness, stability, and discriminative power. Importantly, identifying molecular subtypes through gene expression data provides valuable insights for precision medicine, supporting more personalized and cost-effective treatment strategies.

### Case Study in Testicular Germ Cell Tumor

**Figure 5:**
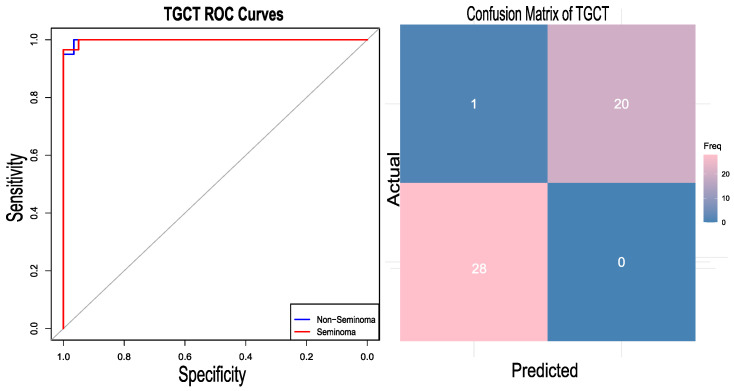
RO curve plotted for the test case dataset of the testicular germ cell tumor (TGCT) and its confusion matrix. The gray diagonal line represents the performance of a random classifier (AUC = 0.5).

The generalization of the developed approach was assessed by applying the same pipeline to a different type of cancer, testicular germ cell tumour (TGCT). Testicular germ cell tumour is considered to be the most common malignancy among men aged between 15 and 35 [41]. Their origin has been hypothesized to be the alteration of primordial germ cells (PGCs) [42]. TGCTs are classified into two categories: seminomas and non-seminomas [43]. Non-seminomas are further divided into embryonal carcinoma (EC), teratoma, yolk sac tumor, and choriocarcinoma. For this case study, the two classes, seminoma and non-seminoma, are considered. The TCGA RNA-Seq dataset [34] was downloaded from the Cbioportal https://www.cbioportal.org/study/summary?id=tgct_tcga_pan_can_atlas_2018 (accessed on 24 August 2024) cBioPortal TGCT Study, which includes two subtypes—seminomas and non-seminomas—as referenced in the same study. The TCGA expression dataset comprises a total of 149 samples; six unlabeled samples were removed from the analysis, and from the remaining samples, 81 are labelled as non-seminomas and 62 as seminomas. SMOTE was utilized to maintain the class balance, yielding 162 data samples. Although the dataset did not have a high degree of class imbalance, oversampling was applied to maintain consistency with the actual pipeline. It should be noted that the results with and without oversampling were not different, suggesting the robustness of the model towards class imbalance. The gene expression data were transformed into the gene set enrichment scores using the *R* package DeepCC. After GSEA analysis, the best 2000 features (gene sets) were selected. The dataset was divided into training and testing sets in a 70:30 ratio. A total of 114 samples were used in the training. Using those 2000 features, the model was trained using the DeepCC classifier. The classifier demonstrated a competent accuracy of 97.96 percent along with an AUC of 99.83 (CI 95%). The heatmap of the confusion matrix and the RO curve analysis are graphically represented in Figure 5.

## 5. Conclusions

Deep learning has demonstrated outstanding potential in various medical applications, including oncology. In this work, a cancer molecular subtype classification model is proposed, distinguished by its integration of feature selection with gene expression data and the use of enrichment scores as input features. Unlike prior approaches, this combination uniquely enhances the model’s ability to identify key molecular characteristics, utilizing fewer features. In particular, the framework demonstrated higher accuracy, sensitivity, specificity, and balanced accuracy, along with consistently strong AUC values, highlighting its generalizability, stability, and discriminative power. Currently, this study focuses on transcriptomic data; integration of multi-omics data and validation across different sequencing platforms are planned for future research. These advances will not only enhance the accuracy of molecular subtyping but also strengthen its clinical applicability.

## Figures and Tables

**Table 1:** The work related to cancer molecular subtyping using different deep learning models is summarized.

Study Reference	Data Type	Approach	Limitation
[16]	Histopathological images, gene expression data, copy number variation (CNV)	CNN for images and a Deep Neural Network for CNV and expression data, combining the results using weighted linear aggregation.	Improved image preprocessing, removing noise, may produce better prediction results. Deep learning models that involve hybrid modality data can be computationally expensive.
[17]	H and E slides	Inception ResNet-based CNN architectures. Separate models were trained for each resolution. The third last layer concatenates all three models for the final prediction.	Multi-resolution models are computationally expensive, and scalability can be a challenge.
[18]	Ultrasound images	ResNet50. To avoid data overfitting, a data augmentation technique was applied to the data, and the stochastic gradient descent was used as the optimizer.	Ultrasound images can be preprocessed to minimize the impact of artifacts.
[19]	RNAseq data and clinical information	The gene expression data were transformed into subtype-specific gene co-expression networks, from which the specific modules were identified.	Integrating the multi-OMIC data may enhance its effectiveness.
[20]	mRNA-seq data, miRNA-seq data, and copy number variation (CNV)	Low-dimensional features were extracted using the denoising autoencoders (DAE), which were used to cluster the subtypes. The logistic regression classification model was built using the clustered subtype data.	The robustness and accuracy of the model are not clearly defined.
[21]	RNAseq data	Pyroptosis-related molecular subtypes were identified using consensus clustering. A classification model was created using CatBoost.	The online databases are used; thus, missing the diversity of patients and response to treatment would lead to non-generalizability.
[22]	Multi-omics data	Extracted the features using the adversarial learning framework, GAN. Used the consensus clustering along with the Gaussian mixture model to identify the molecular subtypes of the tumor samples.	Selecting features diligently from raw input data can lead to better performance.
[23]	Gene expression data and copy number variation (CNV)	Deep CNN models were trained on each data type and then combined in the last layer for the final prediction.	Considering the correlation between different data types during integration can strengthen the model’s validity.
[24]	Whole slides images (WSI)	Designed a model for classifying tumor and non-tumor H and E slide tiles, which was then used to filter out only the tumor tiles for the molecular subtype.	This approach may lack generalizability, as the testing was not performed on external data.
[25]	Gene expression data	Combined CNN and Bidirectional gated recurrent unit	Utilizing genomic data from variable platforms can improve its efficiency.
[26]	Gene expression data	Subtype-specific genes are extracted and then entered into the feedforward NN. L1 and L2 regularization and dropout are used to avoid overfitting	The effectiveness of the proposed framework is highly dependent on the training dataset, limiting its generalisability to unseen data.
[27]	MRI, MET-PET images, and clinical numeric data	Designed a deep neural network using the MRI and patient profile numeric data.	The test accuracy needs to be improved.

**Table 2:** Performance comparison of the DeepCMS(CMS) and DeepCC(CC) models. Acc denotes accuracy, Sens refers to sensitivity, Speci is specificity, and Bal Acc denotes Balanced accuracy.

Dataset	CMS Acc	CC Acc	CMS Sens	CC Sens	CMS Speci	CC Speci	CMS Bal Acc	CC Bal Acc
GSE39582	0.88	0.86	0.87	0.87	0.95	0.95	0.91	0.91
GSE13067	0.92	0.89	0.93	0.87	0.97	0.96	0.95	0.92
GSE37892	0.96	0.93	0.93	0.90	0.98	0.97	0.96	0.94
GSE17536	0.9	0.91	0.87	0.90	0.96	0.96	0.91	0.99

**Table 3:** Comparison of the aggregated efficiency measures, including accuracy, sensitivity, specificity, and balanced accuracy.

Dataset	SVM	Random Forest	DeepCC	DeepCMS
Accuracy	0.90	0.84	0.89	0.92
Sensitivity	0.87	0.84	0.89	0.90
Specificity	0.95	0.94	0.96	0.968
Balanced Accuracy	0.912	0.89	0.92	0.93

## Data Availability

The raw data supporting the conclusions of this article will be made available by the authors on request.

## Abbreviations

The following abbreviations are used in this manuscript:
  TCGA	The Cancer Genome Atlas
  TGCT	Testicular germ cell tumor
  DL	Deep learning
  SGD	Stochastic gradient descent
